# Epiphora and Hyperlacrimation as Paradoxical Manifestations of Facial Nerve Injury: Mechanistic Insights

**DOI:** 10.7759/cureus.59440

**Published:** 2024-05-01

**Authors:** Cadynce Peltzer, Shreya Bhatt, Irene Kamel, Nourdeen Hussini, Yuri Zagvazdin, Mohammadali M Shoja

**Affiliations:** 1 Department of Medical Education, Nova Southeastern University Dr. Kiran C. Patel College of Osteopathic Medicine, Fort Lauderdale, USA; 2 Department of Medical Education, Nova Southeastern University Dr. Kiran C. Patel College of Allopathic Medicine, Fort Lauderdale, USA

**Keywords:** nerve injury, lacrimation, lacrimal gland, facial nerve, bell’s palsy

## Abstract

The incidence of facial nerve paralysis is approximately 30 per 100,000 persons annually. Although it is often idiopathic, as in Bell's palsy, it can also result from infections, trauma, or neoplasms. Facial nerve paralysis may present with partial or total facial paresis, lagophthalmos, denervation of the lacrimal gland, and other ocular abnormalities. While dry eye is a commonly expected outcome of facial nerve injury, some patients may paradoxically experience epiphora and hyperlacrimation. In this review, we examine this phenomenon and its mechanisms in facial nerve injury. Several mechanisms have been proposed for epiphora and hyperlacrimation, including aberrant axonal regeneration, which is known to cause crocodile tears syndrome; ocular irritation due to dry eye, resulting in increased reflex lacrimation due to disruption of the tear film; and impaired drainage of tears caused by paralysis of the orbicularis oculi muscle and malposition of the eyelids. Understanding the pathophysiology of these symptoms is crucial in guiding the management of patients with facial nerve injury. Further experimental and clinical studies focusing on the quantification of tear production and localization of nerve damage will help improve our understanding of the neuroanatomical correlates of this paradoxical manifestation.

## Introduction and background

Facial nerve palsy can result from various causes such as injury, trauma, infection, or neoplasm, or it can be idiopathic in nature, leading to a range of ophthalmological, otological, gustatory, rhinological, and psychological consequences that significantly impact the quality of life [1]. This condition may have either central or peripheral origins, and its inciting lesion may originate from the cortex, temporal bone, the canal of Fallopius, or after the nerve exits through the stylomastoid foramen [2]. The site of the lesion can often be determined by the presence or absence of specific symptoms, heightened sensitivity to sound (hyperacusis), decreased taste sensation in the anterior two-thirds of the tongue, or reduced saliva production and lacrimation [3]. On the affected side, the hemiface appears flat and expressionless, with limited or absent ability to wrinkle the forehead, blink, and grimace [4]. The patient may experience numbness in the affected hemiface, which is a manifestation of an unresolved cortical somatosensory-motor mismatch unrelated to peripheral sensory function [5]. Pain may be present upon touching the external auditory canal and a small patch over the mastoid [4]. Lesions above the stylomastoid foramen lead to reduced salivation and taste sensation (due to loss of chorda tympani function), along with possible hyperacusis. Incomplete or defective eyelid closure (lagophthalmos) can result from paralysis of the orbicularis oculi muscle and/or thixotropy and stiffness of the levator palpebrae superioris muscle, caused by the formation of tight cross-bridges between the actin and myosin filaments of the muscle fibers [6]. Lagophthalmos predisposes the cornea to dry due to accelerated tear evaporation, thereby reducing corneal protection [4,7]. Corneal dryness is further exacerbated by hypolacrimation and reduced tear production due to the loss of presynaptic parasympathetic fibers destined for the lacrimal gland if the nerve lesion is proximal to the geniculate ganglion [3,4].

The discovery of the secretomotor supply to the lacrimal gland is credited to Goldzieher in 1894, who observed that patients with facial nerve palsy often displayed lagophthalmos and reduced tearing on the affected side [8]. While hypolacrimation remains a more common finding, facial nerve palsy can also present with atypical symptoms, including epiphora, defined as abnormal and excessive tearing without a specified cause, and hyperlacrimation, characterized by increased tear production due to hypersecretion by the lacrimal gland [9]. These paradoxical presentations of facial nerve paralysis have been documented since the early nineteenth century. In a review of 250 Bell's palsy cases, it was noted that tear secretion on the paralyzed side exceeded that on the normal side in 11 patients [10]. This underscores the diversity in clinical presentation and raises questions about the pathophysiology underlying decreased versus increased tearing as a result of facial nerve injury. To unravel this complexity, it is essential to outline and comprehend the physiological mechanisms, which serve as the primary focus of this review. We discuss three mechanisms contributing to epiphora and hyperlacrimation in cases of facial nerve palsy: aberrant axonal regeneration leading to the syndrome of *crocodile tears* (also known as Bogarad syndrome or gustatory lacrimation), ocular irritation due to dry eye resulting in increased reflex lacrimation, and reduced drainage of tears caused by paralysis of the orbicularis oculi muscle and malpositioning of the eyelids [9,11,12].

## Review

A brief review of select cases

Several cases spanning over a century to the present day have documented excessive tearing in patients with facial nerve injuries of various origins [13-22]. These cases range from historical accounts, such as a postoperative injury in a young girl [13], to more recent occurrences, including patients experiencing facial nerve injury and/or crocodile tears syndrome following COVID-19 infection [19-22]. Other instances include a 63-year-old male with a three-year history of right facial nerve palsy who despite treatment, continued to experience epiphora [23]. Additionally, there is the case of a 14-year-old female who suffered from co-contracture of the left orbicularis oculi and orbicularis oris muscles, resulting in ectropion and epiphora [14]. Two other examples involve a 39-year-old male with a facial nerve venous malformation that led to lacrimation triggered by olfactory and gustatory stimuli and mastication [16], and a 15-year-old male who experienced hyperlacrimation in the right eye and an inability to close that eye after sustaining minor facial trauma [17]. These reports underscore the fact that hyperlacrimation and epiphora have been observed in individuals of both genders and across all age groups following facial nerve injuries, regardless of the underlying cause. Hyperlacrimation or the accumulation of tears often leads to physical and emotional distress, along with social embarrassment [1,24]. It can also trigger symptoms such as eye irritation and blurred vision [24]. Table 1 summarizes cases of excessive tearing observed in the context of facial nerve injury.

**Table 1:** Summary of cases with excessive tearing in the setting of facial nerve injury.

Author	Age and sex	Etiology of the facial nerve injury	Findings
Fagge, 1904 [13]	Pediatric female	Postoperative (middle ear surgery)	Chronic epiphora
Zaidi et al., 2005 [14]	14-year-old female	Idiopathic	Contracture of the left orbicularis oculi and orbicularis oris muscles resulting in troublesome ectropion and epiphora
Swain et al., 2016 [15]	22-year-old male	Mild head trauma without temporal bone fracture or brain injury	Incomplete closure of both eyes
Rao et al., 2020 [16]	39-year-old male	Facial nerve venous malformation	Lacrimation secondary to olfactory and gustatory stimuli and mastication
Ghimire, 2021 [17]	15-year-old male	Minor face trauma	Inability to close the right eye accompanied by hyperlacrimation
Wang et al., 2021 [18]	2-year-old male	Leukemia with intracranial involvement	Left facial paralysis and incomplete eye closure
Afshar et al., 2021 [19]	64-year-old female	COVID-19 infection	Inability to close the left eye and droopy left eyebrow
Al-Kaisy and Eid, 2021 [20]	58-year-old male	COVID-19 infection	Incomplete closure of the right eye
Iacono et al., 2022 [21]	5-year-old male	COVID-19 infection	Inability to close the right eye
Alrajhi et al., 2022 [22]	56-year-old male	COVID-19 infection	Tearing with eating and synchronous closure of the right eye with jaw movement

Crocodile tears syndrome

**Figure 1:**
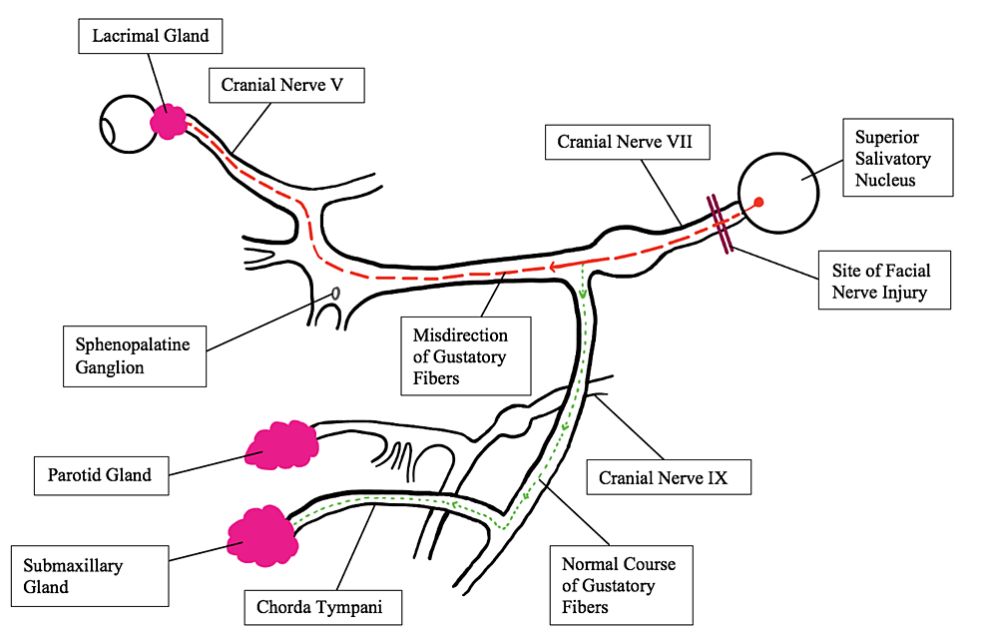
Aberrant nerve fiber regeneration following facial nerve injury. The regenerated postganglionic parasympathetic fibers originally destined for the salivary gland follow the route along the greater superficial petrosal nerve to reach the lacrimal gland. Adapted with some modifications from Montoya et al. [25].

After facial nerve injury, postganglionic parasympathetic fibers originally destined for the salivary gland may undergo rerouting, directing them from the salivary pathway toward the lacrimal gland rather than the submandibular gland. These aberrantly regenerated nerve fibers follow the route along the greater superficial petrosal nerve to reach the lacrimal gland (Figure 1) [12,25].

Consequently, rhinological or gustatory stimuli that would typically induce salivation instead trigger lacrimal gland excitation, leading to tearing. This phenomenon is often referred to as Bogorad syndrome or *crocodile tears syndrome* [11,12]. Crocodile tears syndrome is often a diagnosis of exclusion in individuals with a history of facial nerve palsy that has resulted in synkinesis, a condition arising from the abnormal anatomical reconfiguration of nerves during the healing process [11]. An alternative proposed mechanism for the occurrence of *crocodile tears* is the potential formation of an artificial synapse at the site of injury. In this scenario, impulses propagate from one nerve fiber to another at this artificial synapse, leading to tear formation in response to smelling or tasting food [26].

Reflex tearing

**Figure 2:**
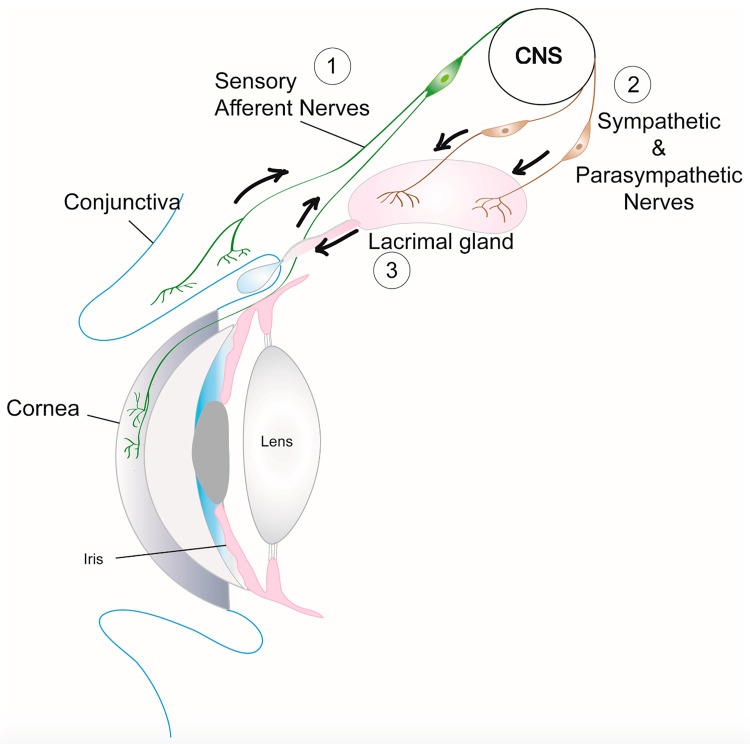
Tear (or lacrimal) reflex. This reflex is mediated by trigeminal afferent fibers that innervate the cornea and conjunctiva and parasympathetic fibers that travel through the facial nerve. Reproduced from Masli and Dartt [29], under Creative Commons license. CNS, central nervous system

The tear film consists of three distinct layers: the inner mucin layer, which adheres to the surface of the eye; the middle aqueous layer, secreted by the lacrimal gland and other accessory glands; and the outer lipid layer produced by the Meibomian and Zeiss glands. The lipid layer plays a crucial role in reducing the evaporation of the aqueous layer at the eye's surface. Disruptions in the layered composition of tears or impediments to eyelid movement that interfere with the composition of the tear film can potentially lead to a reflexive hypersecretion of tears [27]. Reflex tears are generated in greater quantities compared to basal tears and play a crucial role in cleansing the ocular surface by rinsing away irritants [28,29]. Various factors such as trauma, allergies, dry eye, and inflammation can disrupt the functional tear film and trigger tear reflex (Figure 2).

In patients with facial nerve injury, dry eye stimulates the neurosensory receptors present in the cornea and conjunctiva, thereby triggering increased reflex tearing [9]. Several factors, including trauma, allergies, dry eye, and inflammation, have the potential to disrupt the functional tear film and initiate this reflex. In individuals with facial nerve injury, the presence of dry eyes stimulates the neurosensory receptors found in the cornea and conjunctiva, thus provoking an increase in reflex tearing [9]. A dry eye can also promote bacterial growth in the eyelid, which, in turn, intensifies inflammation and worsens the problem of evaporation, thereby establishing a vicious cycle [9].

Paralysis of orbicularis oculi and tear outflow malfunction

In 1957, Lathrop emphasized that facial paralysis, in and of itself, can be just as effective in rendering a soldier unfit for combat duty as wounds that result in tissue loss and pose a threat to life [30]. He pointed out that epiphora, a consequence of facial paralysis when the orbicularis oculi muscle is affected, “prohibits the efficient aiming of small arms" [30]. This underscores the detrimental impact of epiphora in cases of facial paralysis.

**Figure 3:**
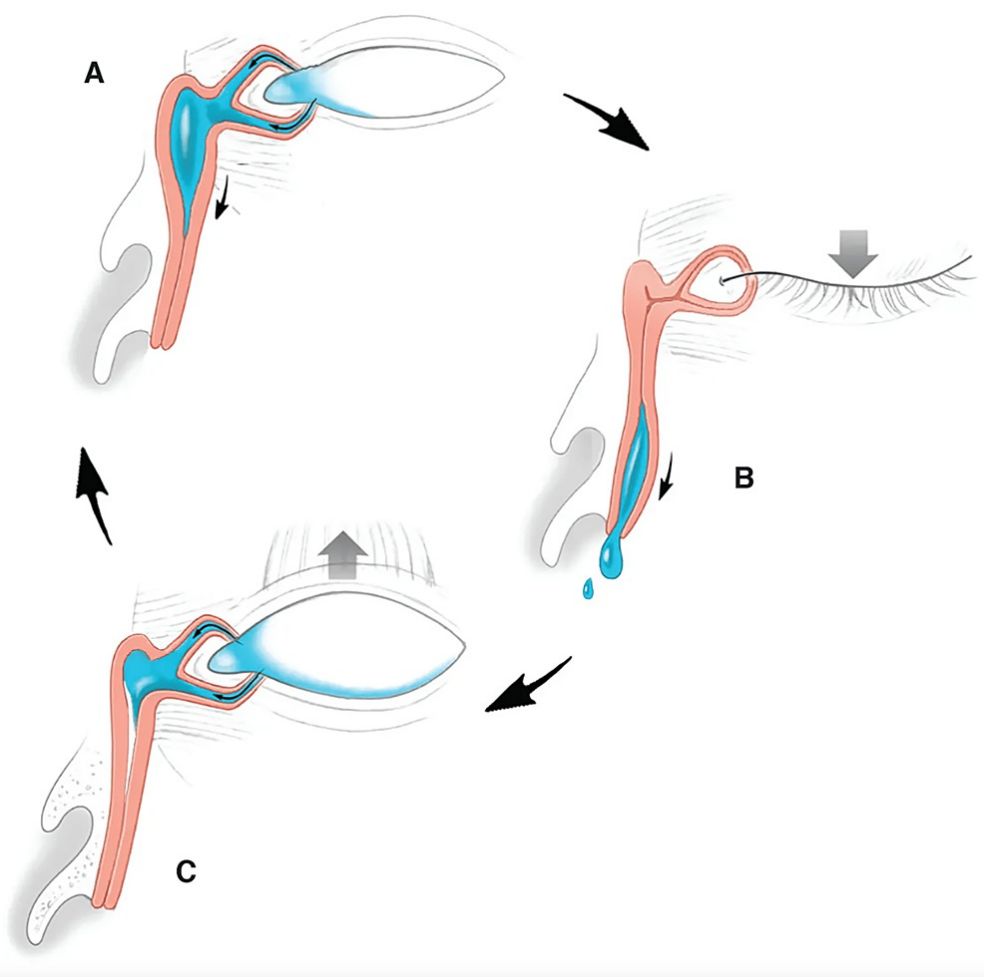
Lacrimal pump. Blinking orchestrates a delicate mechanical cycle, known as the lacrimal pump, to ensure efficient tear drainage. As the eyelids open, relaxation of the orbicularis oculi muscle creates a negative pressure in the lacrimal sac that sucks tears into it (A). Subsequently, as the eyelid closes, the contraction of the orbicularis oculi muscle closes the puncta and canaliculi. The muscle fibers, which also attach to the sac, compress it, facilitating the expulsion of tears through the nasolacrimal duct (B). Finally, reopening of the eyelid with relaxation of the orbicularis oculi muscle allows for the release of pressure on the lacrimal sac (C). This cyclical process repeats with each blink (illustration by Christine Gralapp, reproduced with written permission).

Efficient tear drainage depends on the mechanical cycles created by blinking and the action of the orbicularis oculi muscle on the lacrimal drainage apparatus. Blinking moves tears from the outer corner of the conjunctival sac toward the inner corner [31,32]. Normally, as the eyelids open, the relaxation of the orbicularis oculi muscle serves to dilate and pull forward the lacrimal sac, creating a vacuum-like effect or negative pressure in the sac [31-33]. Simultaneously, the upward movement of the lower eyelid coincides with the punctum moving inwards. Subsequently, as the eyelid closes, the contraction of the pretarsal orbicularis muscle closes the puncta and canaliculi and causes the canaliculi to shorten [9,31]. The muscle fibers, which also attach to the sac, compress it, facilitating the expulsion of tears through the nasolacrimal duct [31]. Figure 3 illustrates the mechanism of the lacrimal pump.

 

To ensure the adequate distribution of tears across the surface of the eye and their passage into the appropriate drainage path, the facial nerve-orbicularis muscle unit functions properly and the eyelids must maintain their normal position, tone, and intermittent blinking. Partial or complete impairment along the blink cycle pathway, responsible for tear circulation, can lead to epiphora [27]. In cases of facial nerve palsy where the orbicularis oculi muscle is either paralyzed or weakened, this pumping mechanism is disrupted [9]. Paralysis of the orbicularis oculi muscle hinders proper eyelid closure, resulting in tear pooling [2]. Reduced lower eyelid tone compromises the ability of the punctum to make contact with the eye globe [10]. Failure of the lacrimal pump hinders the transfer of tears from the conjunctival sac to the lacrimal sac. All these ultimately result in epiphora.

Current treatments

While the optimal management and treatment of acute facial nerve palsy continue to be subjects of debate, it is essential to offer patients all potentially beneficial therapies to prevent the worsening of ocular symptoms, particularly in cases involving impaired lid closure and altered tearing [34]. Currently, the standard treatment involves supportive care. The definitive treatment should be personalized after a careful physical examination to determine which of the above mechanisms is most likely involved in the pathophysiology of excess tearing. In cases of lagophthalmos, transcutaneous or subconjunctival injection of botulinum toxin can induce temporary ptosis and provide corneal protection [35]. Müller's muscle excision (Müllerectomy), levator recession, or myotomy has been utilized to address lid retraction associated with lagophthalmos resulting from thixotropy or stiffness of the levator palpebrae superioris muscle [35,36]. The surgical reduction of the palpebral aperture can alleviate reflex tearing due to dry eye [35]. In some instances, dacryocystorhinostomy may be required to reduce resistance to tear outflow [35]. The administration of botulinum toxin has demonstrated significant improvement in cases of hyperlacrimation resulting from facial nerve injury or Bell's palsy. In a study involving 16 patients experiencing hyperlacrimation, the injection of botulinum toxin into the palpebral lobe of the lacrimal gland yielded promising results. At the one-week follow-up, 11 patients exhibited improved symptoms, and by the end of the first month, all patients no longer experienced hyperlacrimation [37]. Botulinum toxin prevents the release of acetylcholine in the presynaptic membrane thus reducing the production of tears.

## Conclusions

Epiphora and hyperlacrimation associated with facial nerve injury can be attributed to various mechanisms or their combination. These include aberrant nerve regeneration, which results in the *crocodile tears* syndrome, disruption of the tear film leading to corneal irritation and subsequent reflex lacrimation, and impaired tear drainage due to the paralysis of the orbicularis oculi muscle and a faulty pump mechanism. Understanding these key clinical findings and the mentioned mechanisms is crucial for clinicians when selecting therapies and treatment approaches for patients with facial nerve injury. The treatment should be personalized after a thorough physical examination to pinpoint the primary mechanism underlying excessive tearing. The surgical reduction of the palpebral aperture is a valid option for lagophthalmos. An effective therapy for addressing hyperlacrimation is the injection of botulinum toxin into the lacrimal gland, which inhibits the release of acetylcholine at the presynaptic membrane, thereby reducing tear production. It is imperative to conduct further research aimed at expanding the range of available treatment modalities.
